# Overview of dreadful consequences of SARS-CoV-2 invasion in Italy from March 2020 to March 2022

**DOI:** 10.1186/s42269-022-00867-0

**Published:** 2022-06-20

**Authors:** Asma Akter Bhuiyan, Sreyashi Brahmachari, Israt Jahan Ripa, Rashed Noor

**Affiliations:** 1 Molecular Biotechnology and Bioinformatics, Department of Industrial Biotechnologies, Universita Degli Studi di Milano, 20134 Milan, Italy; 2 Medical Biotechnology, Department of Pharmaceutical, Veterinary and Medical Biotechnologies, Università di Bologna, 40126 Bologna, Italy; 3 Department of Life Sciences (DLS), School of Environment and Life Sciences (SELS), Independent University, Bangladesh (IUB), Plot 16, Block B, Bashundhara, Dhaka, 1229 Bangladesh

**Keywords:** Italy, COVID-19 pandemic, Geographical distribution, Infected and death cases, National Health System and Service

## Abstract

**Background:**

The unpredicted pandemic disease COVID-19 first flared up adversely in Europe by imparting interminable force of infected and fatality cases to Italy. In late February 2020, the novel severe acute respiratory syndrome coronavirus 2 (SARS-CoV-2) emerged in northern Italy and swiftly proliferated to the entire country, albeit continuous to date (23 March 2022) with a lesser extent of deadliness. Current review focused on the invasions and the associated consequences by SARS-CoV-2 during the period of March 2020–March 2022.

**Main body of the abstract:**

Initially, the lethality and transmissibility of the novel virus made Italy stunned within 1 month, the number of death cases reached 12,428 at the end of March 2020. The Italian Government announced an immediate emergency phase in entire country, educational institutions to local businesses, manufacturing works, cultural activities to elective activities were rescinded and all the hospitals to morgues were swamped, ensuing that fear of epidemic was impended. Besides, the Italian National Health System and Service coordinated massive public health interventions and conferred unprecedented efforts to limit the high mortality rate of the first wave of infection. Amidst 2 years of epidemic (as of 23 March 2022), Italy has documented 14,070,450 (23.74% of the population) confirmed infected cases, 12,685,306 (21.41% of the population) healed cases, 158,254 death cases (0.27% of the population) and ranking 9th worldwide in the number of deaths.

**Short conclusion:**

Based on publicly available Italian Ministry of Health COVID-19 data, current review has comprehended region-wise total infected cases, death cases and healed cases for three consecutive years 2020–2022 to foresee different patterns of the regional outbreak and gradual subservience. At a glance, we highlighted the overview of the exhaustion and exertion of COVID-19 crisis throughout the periods in Italy.

## Background

At the end of December 2019, numerous people with pneumonia symptoms were admitted to the hospitals of Wuhan, Hubei region of China, and within a short period of time, it spread globally and became a pandemic (Liu et al. 2020). First, “Wuhan pneumonia” was coined to indicate the symptoms; soon after, it was discovered that the causative agent of the disease was “Coronavirus” which belongs to the *“Coronaviridae”* family and β coronavirus genus (Pal et al. 2020; Noor 2021; Noor and Maniha 2020). Subsequently, the name severe acute respiratory coronavirus 2 (SARS-CoV-2) took place (Pal et al. 2020). The World Health Organization (WHO) entitled this pandemic as “COVID-19” disease on March 11 2020, which consists of a genome sequence with an elevated degree of similarity with the earliest epidemic causative virus such as SARS-CoV-1 and MERS-CoV (Noor 2021). To date (19 May 2022), the pandemic had devoured many lives, the number raised to 520,372,492 infected cases and a total of 6,270,232 deaths worldwide (WHO 2022).

Italy was one of the worst afflicted countries during the kick-off of the SARS-CoV-2 massive outbreak and turned into the first European country that abruptly broke the death records after the pandemic mainland, Wuhan, China (Indolfi and Spaccarotella 2020; Maestra et al. 2020). Several studies demonstrated that the SARS-CoV-2 was circulating in Italy along with other countries in Europe, and these cases are supported by epidemiological approaches (Rosa et al. 2021; Wurtzer et al. 2020; Deslandes et al. 2020). Nevertheless, the stream of dead toll was documented as 50% of excess deaths in March 2020 making history of a massive national crisis in Italy after World War II (Indolfi and Spaccarotella 2020; Maestra et al. 2020) and total infected cases had surpassed over 14 million; a total of 14,070,450 (23.74% of the population) cases were enumerated to date (23 March 2022) which indicates the surge of daily increase in cases since the beginning of the pandemic (Istituto Superiore di Sanità 2022; Worldometers 2022). As a consequence, the Italian Government legislated national emergencies, stringent protocols and proclamations including unprecedented lockdown and restriction measures to shut down most of the industrial and commercial activities for containing the epidemic situation (Maestra et al. 2020). Correspondingly, the human life as well as the economic situation of the entire health care service plunged in the blink of an eye (Maestra et al. 2020). Hence, the Act of Italian health system had been a huge trepidation throughout the period of the epidemic.

Servizio Sanitario Nazionale (SSN), Italy’s national health service, was initiated to support the people immediately who were involved in the health sectors and general citizens of the country (Ricci et al. 2020), although there had been some financial mismanagement due to decentralisation and privatisation of health services, which had resulted in a massive negative impact on the health care services and the residents as well (Ricci et al. 2020). Moreover, the contagion made an immense influence on the mental health of the whole Italian individuals according to their age and profession, experienced psychological distress due to the fear of getting infected, loneliness, anxiety and depression due to lockdown and uncertainty of the situation (Rossi et al. 2020; Mansueto et al. 2021). The aim of this study is to confer insights into the manifestations and relevance of COVID-19 in Italy over the time frame of March 2020 to March 2022. Initially, the review enlightens about the earliest manifestations of the SARS-CoV-2 invasion in Italy, followed by a general outline of country's characteristics and the emergence of epidemic in entire Italy and the detailed explanations about the endeavour of Italian health care systems, administrative proclamations and their impact on the entire population. In addition, the study has considered “region” as a geographical unit to support the illustrations throughout the time.

### Data sources

Data for updated COVID-19 cases have traced from Italian National Institute of Health (Ministero della Salute - Istituto Superiore di Sanità) (https://www.iss.it/web/iss-en) (Istituto Superiore di Sanità 2022) and “Worldometer” website (https://www.worldometers.info/coronavirus/country/italy) (Worldometers 2022). We used the regional data till 23 March of three consecutive years 2020, 2021 and 2022 to demonstrate total number of confirmed infected, recovered and deceased COVID-19 cases. To make reasonable comparison, past year’s data have been gathered from “COVID-19 Situazione Italia” (https://opendatadpc.maps.arcgis.com/apps/dashboards/b0c68bce2cce478eaac82fe38d4138b1) (COVID-19 Situazione 2022). To analyse the manifestations of 2-year Italian COVID-19 situation, data have been obtained from the Italian Ministry of Health and Finances (http://www.salute.gov.it) where all national, regional and provincial data are being updated on a daily basis (Ministero della salute 2021; Ministero dell’Economia e delle Finanze 2022; YESMILANO). Italian population records have been found from https://www.statista.com/statistics/617497/resident-population-italy-by-region/ (Statista 2021), and Global data are sourced from “WHO” website (https://covid19.who.int/) (World Health Organisation 2022).

## Main text

### First itinerary of SARS-CoV-2 transmission in Italy

The earliest manifestations of SARS-CoV-2 invasion were initiated in Italy on 23 January 2020 in Rome when a couple of Chinese tourists were flying from Wuhan, fell ill and later admitted to Rome's Spallanzani Hospital with the symptoms of COVID-19 (Giovanetti et al. 2020). Although it was theorised that pathogenic SARS-CoV-2 had been silently circulating in Italy before this first case was spotted, later on, the presence of SARS-CoV-2 was found in northern Italy by analysing several sewage samples and explicit time was before the early period of the epidemic (Rosa et al. 2021). However, as a consequence of two confirmed infected cases, the country announced a “Public Health Emergency” to the entire state for an international concern on 30 January 2020 (Maestra et al. 2020; Giovanetti et al. 2020).

Although the infected Chinese couple was recovered, they maintained mandatory quarantine for 2 weeks and released after the negative serological test results (Putrino et al. 2020). Thereafter, the first autochthonous patient was detected almost 1 month later; on 21 February 2020, a young 38-year-old man was hospitalised in a local hospital of Codogno, a municipality in the province of Lodi, Lombardy region of northern Italy, who unexpectedly never travelled to China (Indolfi and Spaccarotella 2020; Giovanetti et al. 2020; Putrino et al. 2020). However, on the same day, the first COVID-19 death was reported in Italy; another 78-year-old man was hospitalised and demise at Vò Euganeo (Padua), Veneto region of north Italy (Indolfi and Spaccarotella 2020; Bosa et al. 2022). Unquestionably, he was considered the first man of this long string of unexpected deaths (Indolfi and Spaccarotella 2020). Eventually, within 24 h 36 additional cases were documented without any connection to the first infected case (Gastaldelli et al. 2020). Soon after the event, on the 23 February 2020, the Italian government immediately proposed strict measures to contain the spread of the disease and imposed regulations on 11 municipalities within the province of Lodi and put entrance restrictions and announced these spots as “Red Zone” (Bosa et al. 2022). On 25 February 2020, the authority declared a further series of decrees for entire Lombardy and its surrounding regions Veneto, Emilia-Romagna as the outbreak was emerging drastically in Alzano Lombardo and Nembro, two municipalities in the province of Bergamo, Milan (Maestra et al. 2020; Bosa et al. 2022).

Consequently, the Italian Government had enforced progressively more stringent protocols since 28 February 2020 including unprecedented nationwide fractional lockdown to a total lockdown of all non-essential activities, medical interventions, hygienic guidelines, etc. (Bosa et al. 2022; Ghislandi et al. 2020). By March 2020, the surge of SARS-CoV-2-infected cases reached 101,739 and more than 10,000 people demised early in 1 month which made Italy the first severely afflicted country in the whole world (Prezioso and Pietropaolo 2020; Rosa et al. 2021). Within 1 year, the number increased steadily, taking across over 3.5 million infected cases and as of 23 March 2022, the cases surpassed over 14 million, precisely a total of 14,070,450 (23.74% of the population) confirmed cases (Istituto Superiore di Sanità 2022; Worldometers 2022).

### Infected case analysis during March 2020 to March 2022

**Fig. 1:**
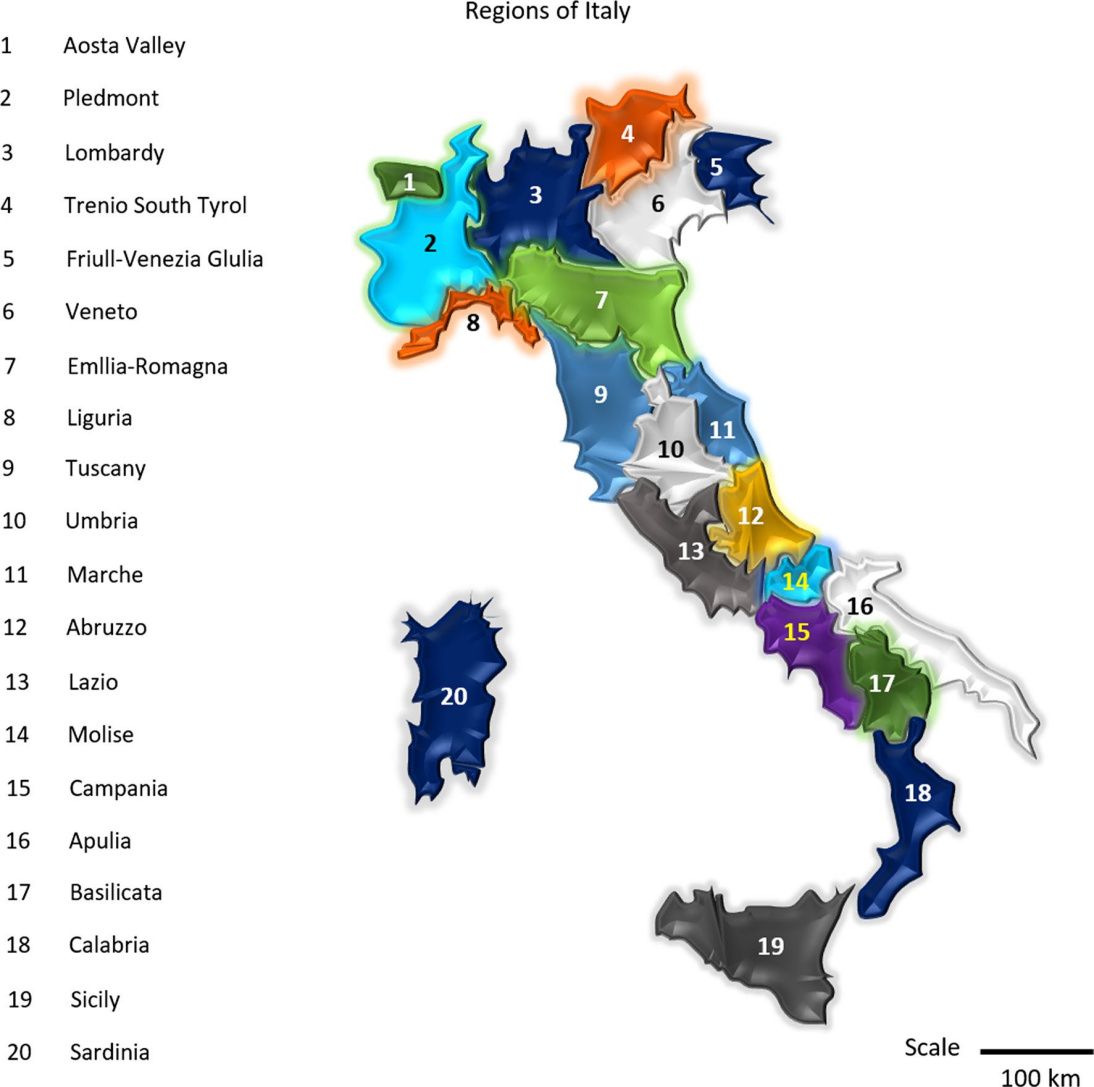
A physical map of Italy showing all the regions of the country

Italy, the boot-shaped peninsula, is situated in southern Europe, which protrudes into the Adriatic Sea, Tyrrhenian Sea, Mediterranean Sea, and other waters (Worldatlas 2022; Ortenzi et al. 2020). The country has owned legitimately 19 regions along with two autonomous regions (equivalent to 1 region) to sustain unique cultural and linguistic differences (Ortenzi et al. 2020; Manica et al. 2021). These regions are broadly considered in five macro-regions for explaining them feasibly, such as the North-West includes Lombardy, Aosta Valley, Liguria, Piedmont; the North-East holds Emilia-Romagna, Friuli Venezia Giulia, Trentino-South Tyrol (P. A Bolzano and P.A Trento) and Veneto. Besides, Sothern and Centre macro-regions enclose the remnant regions, i.e. Lazio, Marche, Tuscany, Umbria, Abruzzo, Apulia, Basilicata, Calabria, Campania, Molise; and the Island contains Sardinia and Sicily (Fig. 1).

Initially, the prevalence of COVID-19 epidemic had been found largely in northern Italy, partially sparing from southern part to the entire country (Bosa et al. 2022). Moreover, to halt the spread of infection in entire regions, the Italian government had created a classification of restrictive tiers, respectively, white (safest), yellow (safer), orange (medium risk) and red (maximum risk) codes. Each colour code indicates several declarations for life safety, strict travel protocols inside the single region and between regions (Gastaldelli et al. 2020). By and large in 2020, the community spread had scattered in different regions in Italy without having any link to the patient 1 and made the situation clueless to estimate the origin of virus in country (Giovanetti et al. 2020; Putrino et al. 2020). However, after 1 month of the first case, Italy has registered in total 63,927 infected cases on 23 March,2020 which had turned to 3,419,616 cases within 1 year as of 23 March 2021 and in the current year of 23 March, the infected cases reached more than 14 million, specifically 14,070,450 cases (COVID-19 Situazione 2022).

**Fig. 2:**
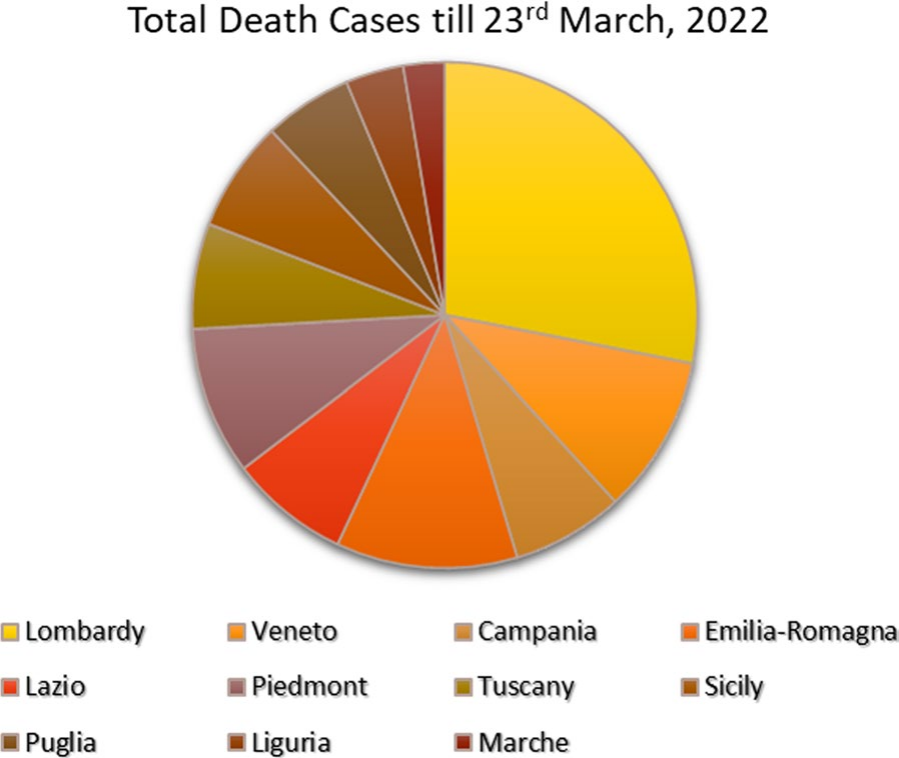
Chart describing the death cases of major regions till 23 March 2022

**Fig. 3:**
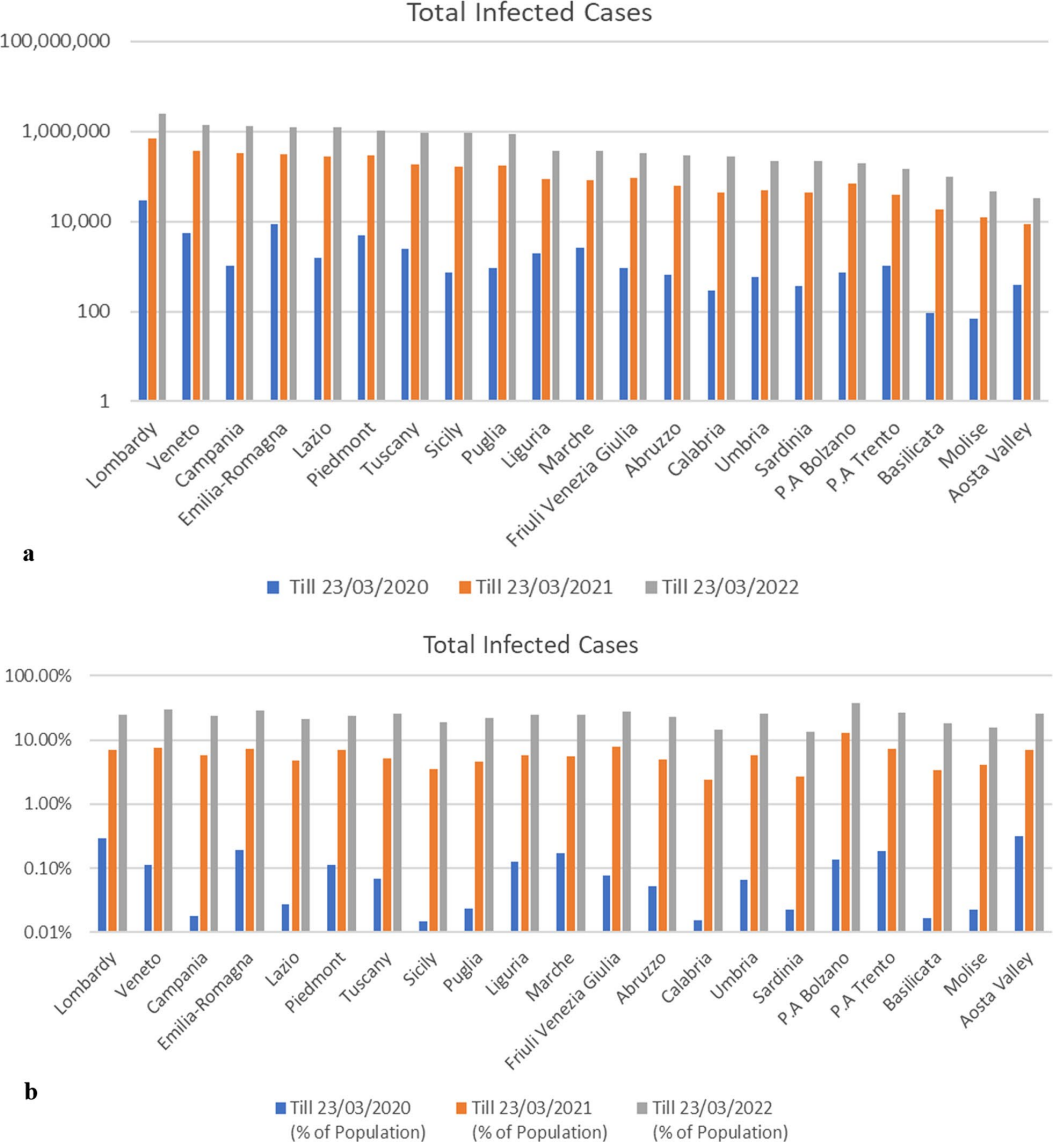
Graphs denoting total COVID-19-infected cases of three consecutive years 2020 (blue lines), 2021 (orange lines) and 2022 (ash lines) of Italy. Data have explained from the first day of viral propagation to 23 March 2020 to 23 March 2021 to the current year of 23 March. Total infected cases are obtained by both molecular swab and rapid antigenic swab tests which is 140,70,450 confirmed positive cases to date. The scheme (**a**) depicts the overall total cases in 20 regions based on the exact number of persons infected by SARS-CoV2. In (**b**) total infected cases have been explained in accordance with total population of each region (% of population)

Lombardy, the largest industrialised region in northern Italy, faced the first wave of COVID-19 phase without any prior symptoms; 3,776 patients deceased only in 2 months. After 1 year, the death cases stood at 29,975, and as of 23 March 2022, it had reached 39,107 cases (Fig. 2) which is 0.39% and 24,070,119 infected cases, which is 24.78% (Fig. 3b) of total inhabitants (9,966,992) of the region. During March 2020, the infected cases reached a high point and continued to May which is considered as the first wave of COVID-19 infection in Italy. Early studies have explained that during the first wave, the infection was spread disproportionately in all regions and related to a high morbidity and mortality (Vinceti et al. 2021; Borghesi et al. 2021).

Therefore, the north-east regions, such as Emilia-Romagna, Veneto and Trento, got the highest hit during the first wave. Besides, north-west regions, Piedmont, Liguria, had been infected with 4861, 1924 cases within 23 March 2020 and these entire Northern regions are considered as the hub of circulating COVID-19 outbreak in Italy. The prevalence of COVID-19 across southern regions, largely on Marche, Tuscany and Lazio with 2569, 2461 and 1540 (Fig. 3a) infected cases, is 4%, 3.8% and 2.4% of total infected cases (63,927) on 23 March 2020. Similarly, other regions of entire territory were transmitted but not sporadically like these regions (Fig. 3a, b); conceivably, early control measures reached these regions before the viral circulation. However, it was undoubtedly clear that thousands of people had infected with the symptomatic and asymptomatic SARS-CoV2 symptoms during the lockdown. Despite the COVID-19 outbreak, some restrictions were drastically alleviated during the summer months of 2020, in terms of both infection rate and disease fatality (Dorrucci et al. 2021). It was considered as “steady transition phase”, as the virus was incessantly mutating to adapt in host and lost much of its lethality (De Natale et al. 2020). Besides, it is likely to reduce due to the notions of seasonal immune response and more germicidal effect of UV rays in summer hot days (De Natale et al. 2020). Nonetheless, the infected cases soared immediately after the summer months and Italy along with other European countries confronted the second wave of the pandemic (Ortenzi et al. 2020). It was anticipated that Italy faced the second wave during the fall of August 2020 to December 2020, although the magnitude of case rate was more in October and peaked on 13 November 2020 (Borghesi et al. 2021). On 2 December, total infected case surpassed 1.6 million, specifically stood at 1,601,554, of which 407,791 cases reported for Lombardy region (Ortenzi et al. 2020; Borghesi et al. 2021). Hence, the region was affected intensely like the first wave of the epidemic. Albeit the infection circulates throughout the regions more proportionately and several studies have shown that younger people were enormously infected in that time, the mortality rate dropped marginally than the first wave (Vinceti et al. 2021; Bongiovanni et al. 2021).

Besides, during the second wave, new variant, i.e. Alpha variant, of COVID-19 was recognised widely along with Italy, which is considered as the most infectious SARS-CoV-2 variant up to now (Dorrucci et al. 2021; Davies et al. 2021; Lai et al. 2021, Noor 2021). Most of the times, the Italian epidemic had uncompromising in terms of both spread rate and lethality (Ricciardi et al. 2020); the total infected cases raised up approximately 54% on 23 March 2021, in entire country. Not surprisingly, Lombardy hold the highest infected case and death cases which has been continued to till March 2022 (Fig. 3a). Though the regions have more inhabitants than other regions which made lower infected percentage in terms of population (Fig. 3b), the autonomous region Bolzano, Trento, that belongs to north-west region has 535,774 and 542,158 inhabitants, respectively (ITALY: Trentino-Alto Adige 2022) and carries a large percentage of infected case rate, approximately 38% and 27% till 23 March 2022, which are greater than other regions (Fig. 3b). Moreover, the prevalence of infected cases arose significantly within 1 year since the start of pandemic and has maintained a constant flow on increase till the current year. At a glance, Northern region [Lombardy, Aosta Valley, Liguria, Piedmont, Emilia-Romagna, Friuli Venezia Giulia, Trentino-South Tyrol (P. A Bolzano and P.A Trento), Veneto] of Italy has afflicted hard throughout the periods with high case number in the population (Fig. 3). On contrary, the community spread is comparatively fewer in southern regions (Lazio, Marche, Tuscany, Umbria, Abruzzo, Apulia, Basilicata, Calabria, Campania, Molise) and in the main islands (Sardinia and Sicily). However, the graphs shown in Fig. 3 clearly explain that overall total cases have significantly declined over the periods.

### Overall case fatality ratio (CFR) in Italy

**Fig. 4:**
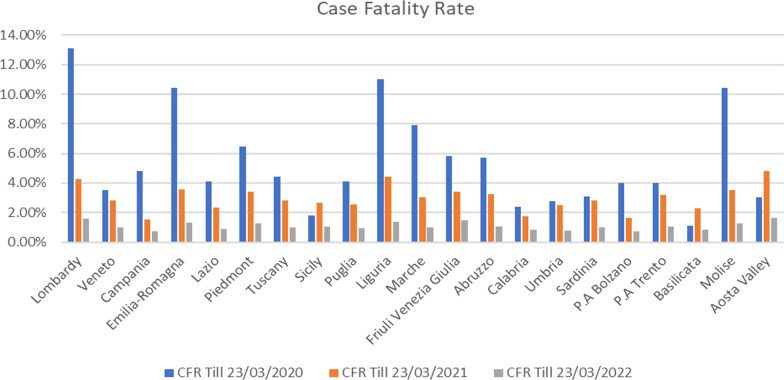
Present scheme compares case fatality ratio (CFR) (percentage of deaths over positive cases was estimated) in three different years (2020, 2021 and 2022) for all regions. It is calculated by the number of deaths attributed to COVID-19 in each region which is divided by the total number of confirmed infected cases of COVID-19 in each region and then multiplied with 100%

Enumeration of case fatality ratio (CFR) has employed to comprehend the trend of epidemic and to arrange essential measurements for curbing the rapid spread of outbreak (Iosa et al. 2020). CFR is defined by the total number of deaths in person caused by the disease and divided by the number of total infected cases in a specific time period. At the beginning of epidemic in Italy, National Institute of Health (Istituto Superiore di Sanità (ISS)) had launched surveillance system to reckon the overall fatality rate of entire country (Gastaldelli et al. 2020). Surprisingly, the fatality ratio of confirmed COVID-19 cases outstripped around 10% within March (9.5% as of 23 March 2020) which was alarming on that time (Onder et al. 2020). This steady increasing trend continued and peaked at 14.52% on June 2020, and over the time it had been slumped significantly within the next 2 years (Fig. 4). In 2020, the overall fatality rate was the highest, whereas it declined drastically in 2021 in all regions. Simultaneously, in the current year the ratio has reached 1.12% in entire country and indicates lower COVID-19 lethality (as of 23 March 2022). Although the ratio was one of the highest during the kick-off epidemic to estimate overall country fatality ratio, this high percentage of CFR was misleading as the ratio does not signify that COVID-19 is more deadly in Italy than elsewhere in the world (Iosa et al. 2020; Onder et al. 2020). Iosa et al. (2020) and a number of studies have reasoned about miscomputation of CFR and explained several methodological discrepancies in case of documenting and testing, i.e. unexpected high number of death cases in northern Italy depicted in Fig. 4, misestimating in testing strategies for symptomatic and asymptomatic patients, etc., and because of these reasons the country lost to enumerate true magnitude of epidemic at the first stage and emergency situation (Onder et al. 2020).

### Estimation of total healed cases over the time period studied

**Fig. 5:**
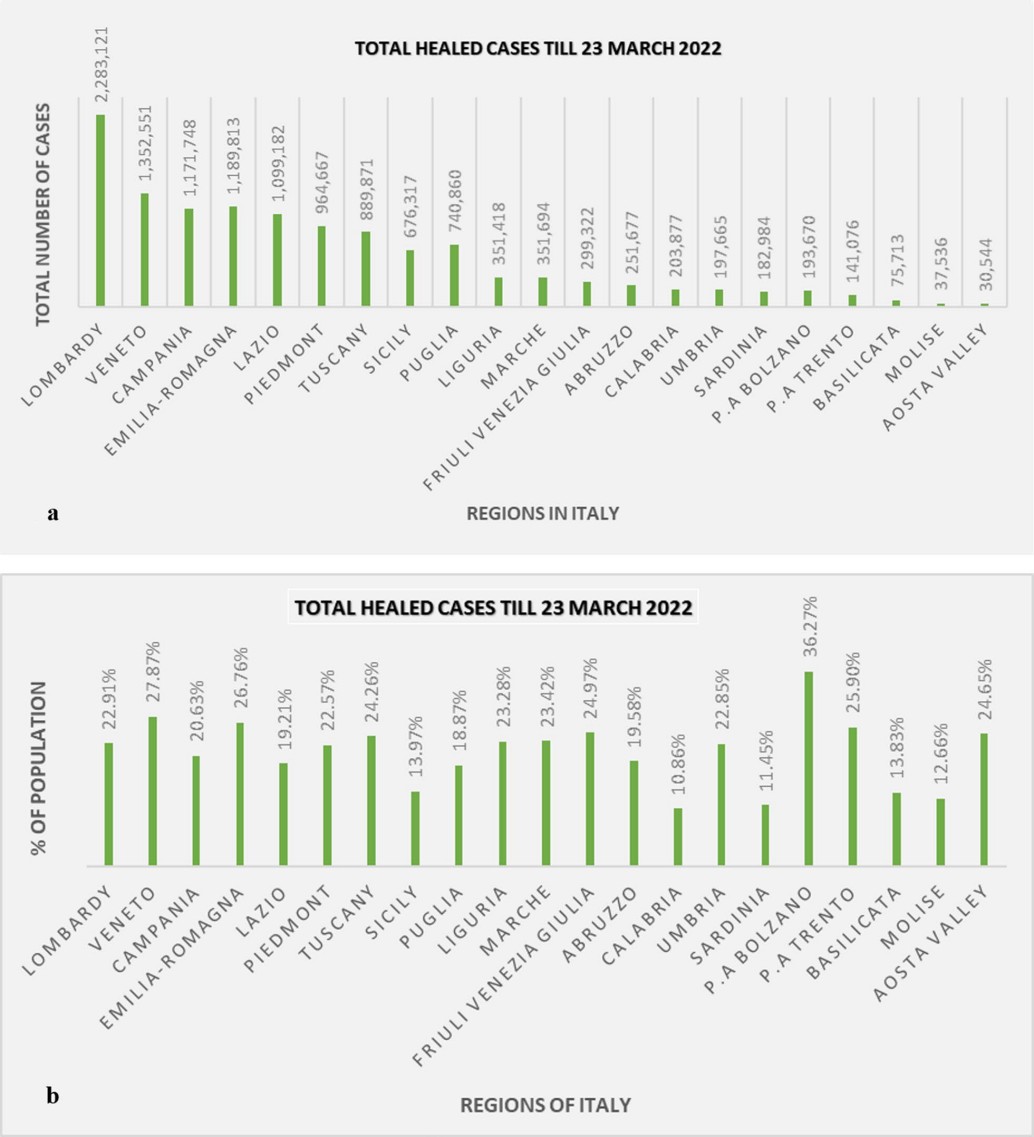
These data address the estimation of total healed cases in all regions throughout the 2 years of period.** a** Number of recovery cases till 23 March 2022;** b** Total recovered cases in accordance with total population of each region of Italy. Details are given in the text

Since the starting of COVID-19 epidemic, the exact parameter of the disease was unreliable and different symptoms of complexities, mostly hypertension (69%), type-2 diabetes (32%), chronic renal failure (21%), ischaemic heart disease (27%), etc., were considered for the excess mortality of COVID-19 infection (Onder et al. 2020; Michelozzi et al. 2020). Although the recovered patient number had escalated and surpassed the overall death cases of the pandemic year, soon after, the recovered cases have reached nearly 12.7 million, specifically 12,685,582 cases till 23 March 2022, which is more than 80% of total infected cases (Worldometers 2022). Noticeably, all the Italian regions have exhibited dramatic variations in local outbreaks to recovered cases over the periods. To date (23 March 2022), most of the patients have recovered in Lombardy region, 2,283,121 cases that indicates 22.91% inhabitants of the region, though the percentage is smaller than other northern regions (Fig. 5). The scheme shown in Fig. 5a directly presents the number of recovery cases till date (23 March 2022); the northern east and west regions such as Lombardy, Emilia-Romagna, Friuli Venezia Giulia, Trentino-South Tyrol (P. A Bolzano and P.A Trento), Veneto and Piedmont, Liguria and Aosta Valley have the largest number of recovery cases than the southern and central regions. In Fig. 5b, the total recovered cases have been explained in accordance with total population of each region (% of population). Briefly, entire Italy has substantially made more healed cases than death cases by regulating the situation. However, the overall highest percentage of inhabitants have been recovered in all north-eastern regions such as Emilia-Romagna, Veneto, Friuli Venezia Giulia, P. A Bolzano and P.A Trento, respectively, 26.76%, 27.87%, 24.97%, 36.27% and 25.90% (Fig. 5b).

Besides, the autonomous region Bolzano has total 193,670 recovered patients which indicates the most significant percentage of inhabitants (533,715), almost 37% are recovered to date along with other regions (Fig. 5b). Similarly, the southern and central region has been improving in the recovered cases, except a decrease percentage of 10.86% that occurred in Calabria with 203,877 recovered cases (Fig. 5b). Comparatively, island regions such as Sicily and Sardinia have accounted large number of inhabitants and less recovered cases; hence, the percentage stands at roughly 14% and 11.5% (Fig. 5b). On the whole, pandemic continues to recede, and the healing statistics is improving gradually within these years and will tend to increase inevitably day by day.

### Italian health care system and their exertion throughout the infectious period

National Health System (NHS) of Italy, also known as Servizio Sanitario Nazionale (SSN), is one of the finest health care systems among all the countries in the whole world. It holds the second position after France, based on the analysis of the structure and overall performance of the health systems, by WHO on 2000 (Coyne and Hilsenrath 2002). SSN was introduced to the Italian citizens on 23 December 1978 by the Ministry of Health and also supported by various health care agencies (Falco 2019; Ferre et al. 2014). This tax-dependent health care system, acknowledged by WHO and OECD (Organisation for Economic Co-operation and Development), relies on fundamental principles and ethics such as providing a uniform access of health services to every citizen irrespective of their income or location, developing productive schemes effective against disease prevention, monitoring health expenditure and giving public democratic control (Rechel 2018; Ministero della salute 2021). Moreover, SSN is regionally organised health system which is represented as a manifestation of human dignity, universality, proper health requirements and solidarity (Rechel 2018).

In Italy, the Central Government has been responsible for the administrative section of health care which includes universal, standard health services and uniformity of access by introducing a crucial health care benefit package, known as *Livelli Essenziali di Assistenza* (LEA) (Bosa et al. 2021), while regional governments have taken account of organising and providing health care depending on the ideas stated at the national level with the help of their own health departments and a network of local health authorities based on population called *Aziende Sanitarie Locali* (ASLs). These organisations are completely autonomic which provide public and community health assistances directly or with the aid of public hospitals and authorised private providers (Ferre et al. 2014). However, these decentralisation and localisation of the national health care systems have weakened the financial strategy of the Italian government. During the period 2010–2019, more than €37 billion were curtailed from the National Healthcare Service budget (Armocida et al. 2020).

After the first COVID-19-infected case declared on February 2020, the SSN started facing an increasing pressure, with massive number of infected cases and early deaths (Gatto et al. 2020). The deep-rooted financial predicament of public health services had gradually weakened the NHS system which almost collapsed to manage such a difficult situation (Ricci et al. 2020). Since the severity of the disease started to elevate, it became really hard to cope with the increasing cases of hospitalisations of COVID-19 patients. Thus, availability of general beds along with intensive care beds tended to decrease and the health professionals were obligated to work for more than their prescribed hours (Vicentini et al 2022). Not only that, due to the lack ability of the NHS, patients were treated depending on their severity, giving priority to the patients requiring immediate respiratory support (Michelozzi et al. 2020). Besides, most of the health care workers had suffered from symptoms like weakness, fever, cough due to constant exposure to this contagious infection without any adequate amount of self-protected equipment such as PPE, surgical masks and sanitizers (Mansueto et al. 2021; Vicentini et al. 2022). In addition, few studies have published that the health worker acquired serious mental health problems like anxiety, depression, sleep disturbances and high levels of stress due to COVID-19 risk factors along with extreme work pressure and anger for the unexpected pandemic scenario as well as the unawareness of NHS system (Mansueto et al. 2021; Vicentini et al. 2022, De et al. 2021).

### Government aid and proclamations

To avert the limitations of NHS, the Government of Italy tried to mend the situation by arranging some essential resources and finances for the regions which were affected mostly due to the pandemic. For instance, Lombardy region was provided with 3800 respiratory ventilators, 30 million face protection masks and 67,000 tests for detecting SARS-CoV-2 by an advanced public procurement under Italian Civil Protection, in addition to 20,000 health workers and €660 million issued by Italian Government (Armocida et al. 2020). Italian government issued a number of decrees in order to support the general population including people involved in health care services (Armocida et al. 2020; Ricci et al. 2020). Among them, the most significant ones were “Cura Italia” and “liquidity”.

“Cura Italia” was the first ordinance released on 17 March 2020 which assigned 150 million funds to health sectors for remunerating doctors and nurses for their contribution to the society (KPMG 2022). Likewise, individuals having Master’s degree in medicine and surgery were allowed to practice of the medical profession, so that they could back up other doctors during the crisis of staffs and nurses. Also, taxes for the sanitation of common areas such as workplaces were excluded, and office employees were given bonuses to support their family in pandemic situation (Ricci et al. 2020). Even to support working parents, Government started a coupon worth up to € 600 for babysitting services (Ministero dell’Economia e delle Finanze 2022). Eventually, “Liquidity”, the freelancer doctors and physicians were provided £600,00 from the state income deposit as their income was drastically declined due to the hospitalisations. Financial aids were given to families and some companies via Central Guarantee Fund (CGF). Apart from that, individuals like businessmen, teachers and persons involved in other professions, whose income was below 2 million, and people belonging to “red zone” area were exempted from paying taxes (Ricci et al. 2020). Besides, to rectify and strengthen these decrees, Relaunch and August decrees were further declared that introduced new schemes to give support parents, disabled members and students by providing extra resources, funds and assistance (Ministero dell’Economia e delle Finanze 2022). In addition, the government had launched different technological infrastructure, such as mobile app “immune” to introduce a surveillance system for maintaining contact tracing or social distancing, giving vaccination procedures, etc., for all regions (Bosa et al. 2022).

### Lockdown and social distancing in Italy

To date, social distancing is considered to be the finest measure to defend and control horrific situation of infectious COVID-19. In Europe, the Italian government first executed the nationwide lockdown which continued almost 2 months (from 8 March to 4 May 2020) to contain the contagion (Eichenberg et al. 2021). Ensuing, people movements were strictly constricted to go maximum risky areas (inter-regional mobility), banning migration from one to other municipalities except health workers or emergency situations, suspending all sort of non-essential, retail/recreational activities, and ceasing access to public places like parks, schoolyards and gardens, etc. (Manica et al. 2021; Cena et al. 2021). And, due to the rigorous restrictions and selfless effort of doctors and health care workers, the number of hospitalisation cases and patients started to reduce slowly. Lately, after 4 May 2020 Italy moved into “second phase” of lockdown where the previous restrictions were alleviated, and slightly relaxed regulations and safety measures were reflected (Eichenberg et al. 2021). For instance, keeping a certain physical distance (1 m) between two persons, proper isolation and RT-PCR test activity were allowed to ease the lockdown and resume the normal daily lives of people by reopening schools and offices, etc. (Cena et al. 2021).

However, withdrawing and easing the regulations made false effects on citizens and Italy entered into second wave of epidemic at the end of 2020 (Manica et al. 2021; Dorrucci et al. 2021). Nevertheless, the government escalated the protection measurements, made strict to follow and introduced a three-tiered restriction system (coded as yellow, orange and red) in each of the 21 regions to measure epidemiological risk assessment (Manica et al. 2021). The system was launched on 6 November 2020, and as of May 2021, the tier has continued with legislated restrictions on different regions and considered as fundamental approach to handle the region-wise SARS-CoV-2 infections before the vaccination campaigns (Manica et al. 2021). After proceeding in this way till date, the mortality rate started to drop and came to a stable point considering the positive effectiveness of the strict measurements (Auriemma and Iannaccone 2020).

### Vaccine recognition and “Green Pass”

The concept of green pass was solely proposed by the European Commission for the free movement of citizens in the European Union that has been executed in Italy on 17 June 2021 by Italian Health Minister (EU digital COVID certificate 2022). Green pass or EU digital COVID certificate (also known as SARS-CoV-2 Green Certification) is a digital as well as printable certification, which contains a two-dimensional barcode (QR Code) and a qualified electronic seal for authentication (EU digital COVID certificate 2022). First, it was issued to the individuals who completed two basic courses of vaccines; lately, a *super* Green Pass has been issued for fully vaccinated persons with a full course (booster dose) of an approved vaccine (Ministero degli Affari Esteri 2022). From 23 September 2021, the Italian Ministry of Health approved vaccines which are recognised by WHO and equivalent officialdoms to prevent SARS-CoV-2, namely vaccines recognised by EMA—European Medicines such as Pfizer-BioNtech Comirnaty, Moderna, Vaxzevria, Janssen (Johnson & Johnson), Nuvaxovid (Novavax), Covishield, R-CoVI (R-Pharm), COVID-19 vaccine recombinant (Fiocruz) which are manufactured under licence from AstraZeneca (Kaushal & Noor 2022; Noor et al. 2022; Ministero degli Affari Esteri 2022).

As of 23 March 2022, 50,712,944 persons (84% of the total population) have been administrated with at least one dose of vaccine and 47,781,637 (79% of the population) with complete doses of vaccine (WHO 2022). However, the vaccine has been exempted in some criteria, alike minority such as all children below 6 years of age, transport crew members, on board transport staff, cross-border workers, etc. Apart from the exceptions, the pass and FFP2 mask are obligatory for all distinct inhabitants to access all the services, outdoor places and public transports (YESMILANO).

### Economic impression in Italy during the pandemic

At the beginning of the outbreak in China, Italy halted all the flights that travel to or from China following with a check-up of temperature and quarantine for 14 days if there was any contact with the COVID-19 patients (Auriemma and Iannaccone 2020; Shang et al. 2021). Besides, during the first wave of lockdown, as the excess mortality rate was uncontrollable, the Italian government consequently provided health facilities for patients with COVID-19-related issues and neglected other issues which lead to the downfall of the economic growth, livelihood and social system by interruption in business markets, manufacturers, prohibition of travel, shutdown of cities, etc. (Auriemma and Iannaccone 2020). Although Italy economy is one of the highest ranks in the world, the lockdown period emphasised hardship of economic sectors and particularly on tourism sector. Italy owned world’s famous tourist destinations, and the economic upshot made the gross domestic product (GDP) slump along with unemployment in the sector. In the tourism sector, unemployment raised to a number of 215,000 in 2020, which contributed to 12.4% of employment; apart from that, in Italy there was a loss of 121 billion euros in the economical tourism sector because of COVID-19 (Travel Tomorrow 2022).

Owing to the lockdown situation and being the most affected country in 2020, Italy's GDP fell by approximately 8.87 percentage than past years. After a drastic decrease in the economy, GDP raised to 5.77% in 2021 and 4.23% in 2022 as per previous years (Statista 2022). COVID-19 restrictions managed individuals to work remotely in the reopening stages and promote Italy to digitalisation, specifically public service sector and education sector; also till date (March 2022) remote system is conducted (Taglietti et al. 2021). Besides these sectors, agricultural, manufacturing goods, industrial, hotels, bars and restaurants are the major contributors to GDP growth. Currently, Italy has succeeded to overcome the crisis; moreover, gradually the COVID-19 restrictions are eliminated by the officials, manufacturing goods, industries and tourism sectors and therefore commenced to become normalised (Auriemma and Iannaccone 2020).

## Conclusions

The world has experienced various infectious disease outbreaks throughout history, and among them, the COVID-19 pandemic has endowed a massive impact worldwide (Noor and Maniha 2020). Italy was the first European country which got the highest number of death cases immediately after China and abruptly confronted the frightening effect of COVID-19. During the first wave, the transmission chain of infectious cases was disproportionate in all regions, while it became more proportionate in the second wave, albeit the northern regions experienced the most severe effect of the pandemic throughout the period. This systematic review has shed a light on the overall effect of the pandemic in terms of health and economy of Italian citizens. With the proclaimed containment measures by the Italian government and the enormous effort of Italian health system, health care professionals minimised the number of early demised cases. Since March 2022, the fight against the SARS-COV-2 has been perilous to cope with the situation. Nevertheless, after 2 years of agony, on 24 March 2022, health minister Roberto Speranza announced a final decree regarding the exemption of the emergency phase which has been going on in Italy (Ministero degli Affari Esteri 2022). According to the new decree, Italy will enter a new phase from 1 April and the decree has included some formal instructions for individual classes of citizens, for instance, the Digital Passenger Locator Form, the COVID-19 Vaccine report for arriving in Italy, etc. Besides, mandatory Super Green Pass for everyone over the age of 12 as a valid proof of vaccination or recent recovery from COVID-19, which helps to access most hospitality venues, public transport and activities (ITALY GREEN PASS 2022). Significantly, from 1 May 2022, the Green Pass will no longer be required and all other activities in kindergartens, primary, secondary schools along with other educational institutes will be in the presence of proper restrictions (YESMILANO, Ministero della Salute 2022). However, those who have interacted with a COVID-19-infected person will have to wear FFP2 mask for 10 days from the last contact and the infected person will have to be isolated at home (Ministero della Salute 2022).

Although having a huge negative effect in general, this pandemic unknowingly helped in improvement in science and technological fields. Several novel technologies such as artificial intelligence, open-source technologies and many more were introduced to fight against the virus which led to an uplift of the scientific arena (Kritikos 2020). The latest evidence in our literature review suggests that all the implemented rules and stringent protocols are finally able to diminish the severity of the infection, indicating that the emergency phase is almost over and finally people have got a ray of hope to get back to the usual life before this unpredicted pandemic situation.

## Data Availability

Not applicable.

## Abbreviations

ASL: Aziende sanitarie locali
CFR: Case fatality ratio
CGF: Central Guarantee Fund
EMA: European Medicines Agency
EU: European Union
FFP2: Filtering face-piece 2
GDP: Gross domestic product
ISS: Istituto superiore di sanità
LEA: Livelli essenziali di assistenza
MERS-CoV: Middle East respiratory syndrome coronavirus
NHS: National Health Service
OECD: Organisation for Economic Co-operation and Development
P.A Bolzano: Provincia autonoma di Bolzano (Autonomous Province)
P.A Trento: Provincia autonoma di Trento (Autonomous Province)
PPE: Personal protective equipment
RT-PCR: Real time polymerase chain reaction
SARS-CoV-2: Severe acute respiratory syndrome coronavirus 2
SSN: Servizio sanitario nazionale
UV: Ultraviolet
WHO: World Health Organization
